# *N*-(2-Formyl­phen­yl)benzene­sulfonamide

**DOI:** 10.1107/S1600536809032681

**Published:** 2009-08-22

**Authors:** S. Thenmozhi, S. Ranjith, A. SubbiahPandi, V. Dhayalan, A. K. MohanaKrishnan

**Affiliations:** a Department of Physics, Presidency College (Autonomous), Chennai 600 005, India; b Department of Organic Chemistry, University of Madras, Guindy Campus, Chennai 600 025, India

## Abstract

In the title compound, C_13_H_11_NO_3_S, the two aromatic rings are oriented at an angle of 88.18 (8)°. Intra­molecular N—H⋯O and C—H⋯O hydrogen bonds are observed, each of which generates an *S*(6) ring motif. In the crystal, mol­ecules are linked into *C*(7) chains along [010] by inter­molecular C—H⋯O hydrogen bonds. The structure is further stabilized by inter­molecular C—H⋯π inter­actions involving the sulfonyl-bound phenyl ring.

## Related literature

For the biological activity of sulfonamides, see: Zareef *et al.* (2007 ▶); Chohan *et al.* (2007 ▶); Brown (1971 ▶); Pomarnacka & Kozlarska-Kedra (2003 ▶); Sethu Sankar *et al.* (2002 ▶). For related structures, see: Bassindale (1984 ▶); Cotton & Stokley (1970 ▶); Usha *et al.* (2005 ▶); Zhu *et al.* (2008 ▶). For hydrogen-bond motifs, see: Bernstein *et al.* (1995 ▶).
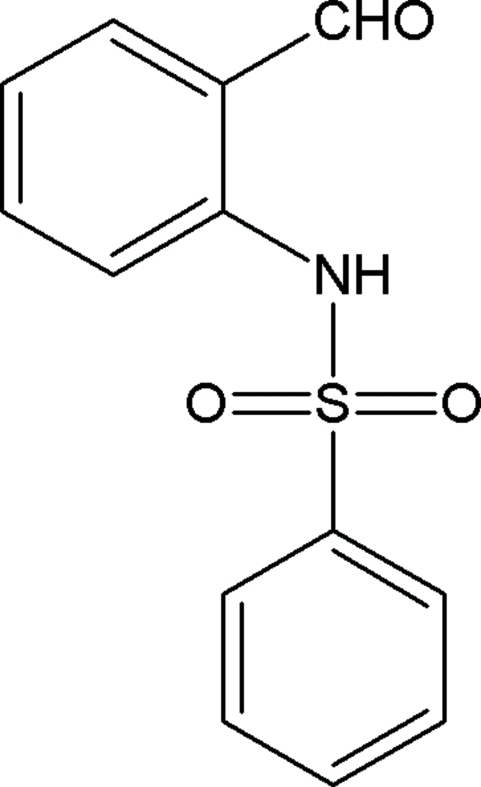


## Experimental

### 

#### Crystal data

C_13_H_11_NO_3_S
*M* *_r_* = 261.29
Triclinic, 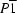

*a* = 7.7656 (2) Å
*b* = 9.0080 (2) Å
*c* = 9.5855 (2) Å
α = 86.293 (1)°
β = 77.912 (1)°
γ = 68.826 (1)°
*V* = 611.35 (2) Å^3^
*Z* = 2
Mo *K*α radiation
μ = 0.26 mm^−1^
*T* = 293 K
0.21 × 0.19 × 0.17 mm

#### Data collection

Bruker Kappa APEXII CCD diffractometer
Absorption correction: multi-scan (*SADABS*; Sheldrick, 1996 ▶) *T*_min_ = 0.768, *T*_max_ = 0.956
15490 measured reflections
3960 independent reflections
3228 reflections with *I* > 2σ(*I*)
*R*_int_ = 0.022

#### Refinement

*R*[*F* ^2^ > 2σ(*F* ^2^)] = 0.040
*wR*(*F* ^2^) = 0.119
*S* = 1.02
3960 reflections
167 parameters
H atoms treated by a mixture of independent and constrained refinement
Δρ_max_ = 0.31 e Å^−3^
Δρ_min_ = −0.31 e Å^−3^

### 

Data collection: *APEX2* (Bruker, 2004 ▶); cell refinement: *SAINT* (Bruker, 2004 ▶); data reduction: *SAINT*; program(s) used to solve structure: *SHELXS97* (Sheldrick, 2008 ▶); program(s) used to refine structure: *SHELXL97* (Sheldrick, 2008 ▶); molecular graphics: *ORTEP-3* (Farrugia, 1997 ▶); software used to prepare material for publication: *SHELXL97* and *PLATON* (Spek, 2009 ▶).

## Supplementary Material

Crystal structure: contains datablocks global, I. DOI: 10.1107/S1600536809032681/ci2869sup1.cif

Structure factors: contains datablocks I. DOI: 10.1107/S1600536809032681/ci2869Isup2.hkl

Additional supplementary materials:  crystallographic information; 3D view; checkCIF report

## Figures and Tables

**Table 1:** Hydrogen-bond geometry (Å, °)

*D*—H⋯*A*	*D*—H	H⋯*A*	*D*⋯*A*	*D*—H⋯*A*
N1—H1⋯O3	0.80 (2)	1.99 (2)	2.6751 (19)	144 (2)
C2—H2⋯O1	0.93	2.46	3.0879 (18)	125
C3—H3⋯O2^i^	0.93	2.56	3.2691 (19)	133
C5—H5⋯*Cg*1^ii^	0.93	2.80	3.700 (2)	162

Symmetry codes: (i) 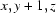; (ii) 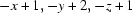. *Cg*1 is the centroid of the C8–C13 ring.

**Fig. 1.**
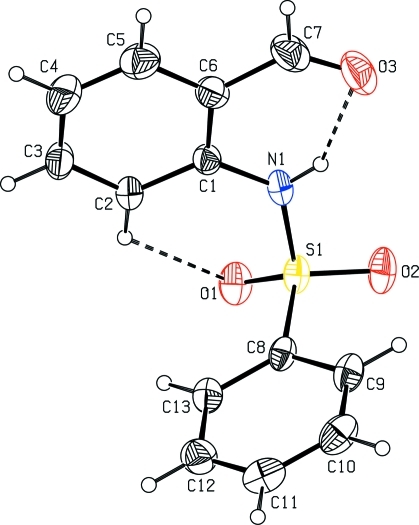
The molecular structure of the title compound, showing 30% probability displacement ellipsoids and the atomic numbering scheme. Hydrogen bonds are shown as dashed lines.

**Fig. 2.**
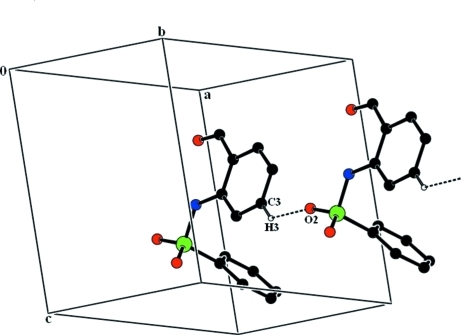
Part of a C(7) chain in the title compound.

## supplementary crystallographic information

### Comment

Sulfonamides have been recognized for their wide variety of pharmacological activities such as antibacterial, antitumor, anti-carbonic anhydrase, diuretic, hypoglycaemic, antithyroid and protease inhibitory activities. Sulfonamides particularly sulfadiazine and sulfadoxine have been used clinically as antimalarial agents (Zareef *et al.*, 2007). Due to their significant pharmacological applications and widespread use in medicine, these compounds have also gained attention in bioinorganic and metal-based drug chemistry (Chohan *et al.*, 2007). Sulfonamide derivates are well known drugs and are used to control diseases caused by bacterial infections. The antibacterial action of this group of drugs is exerted by the complete inhibition of dihydropteroate synthase enzyme towards the *p*-amino benzonate (Brown, 1971). Benzene sulfonamide derivatives are known to exhibit anticancer and HIV activities (Pomarnacka & Kozlarska-Kedra, 2003). The sulfonamides inhibit the growth of bacterial organism and are also useful for treating urinary and gastrointestinal infections (Sethu Sankar *et al.*, 2002). In view of this medicinal importance, the crystal structure determination of the title compound (Fig.1) was carried out and the results are presented here.

Atom S1 has a distorted tetrahedral configuration. The widening of angle O1—S1—O2 [119.76 (7)°] and narrowing of angle C8—S1—N1 [106.08 (6)°] from the ideal tetrahedral value are attributed to the Thorpe-Ingold effect (Bassindale, 1984). The two aromatic rings are oriented at an angle of 88.18 (8)°. The angles around atom S1 deviate significantly from the regular tetrahedral value, with the largest deviation of 119.7 (7)° for O1—S1—O2 angle. This may be due to non-bonding interactions between S—O bonds (Cotton & Stokley, 1970). The aldyhyde group is coplanar with the benzene ring, as evidenced by the torsion angle C1—C6—C7—O3 of -2.2 (3)°. The geometrical parameters agree well with those reported for related sulfonamide structures (Usha *et al.*, 2005; Zhu *et al.*, 2008). Intramolecular N1—H1···O3 and C2—H2···O1 hydrogen bonds are observed, each of which generates an S(6) ring motif (Bernstein *et al.*, 1995).

Intermolecular C—H···O hydrogen bonds involving atoms C3 and O2 link molecules into C(7) chains running along the [010] (Fig. 2). The crystal packing is further stabilized by C—H···π interactions involving the sulfonyl-bound phenyl ring.

### Experimental

*N*-[2-(Hydroxymethyl)phenyl]benzenesulfonamide (2 g, 7.6 mmol) was added to a solution of pyridinium chlorochromate (3.25 g, 15.11 mmol) in dry dichloromethane (20 ml) at room temperature and the reaction mixture was stirred for 4 h. The solvent was removed to obtain a crude aldehyde as a white solid. Single crystals of the title compound suitable for X-ray diffraction were obtained by slow evaporation of a methanol solution.

### Refinement

Atom H1 was located in a difference map and refined freely. All other H atoms were positioned geometrically and constrained to ride on their parent atoms, with C-H = 0.93 Å and U_iso_(H) = 1.2U_eq_(C).

### Crystal data

**Table:**

C_13_H_11_NO_3_S	*Z* = 2
*M**_r_* = 261.29	*F*(000) = 272
Triclinic, *P*1	*D*_x_ = 1.419 Mg m^−^^3^
Hall symbol: -P 1	Mo *K*α radiation, λ = 0.71073 Å
*a* = 7.7656 (2) Å	Cell parameters from 3960 reflections
*b* = 9.0080 (2) Å	θ = 2.2–31.3°
*c* = 9.5855 (2) Å	µ = 0.26 mm^−^^1^
α = 86.293 (1)°	*T* = 293 K
β = 77.912 (1)°	Block, colourless
γ = 68.826 (1)°	0.21 × 0.19 × 0.17 mm
*V* = 611.35 (2) Å^3^	

### Data collection

**Table:**

Bruker Kappa APEXII CCD diffractometer	3960 independent reflections
Radiation source: fine-focus sealed tube	3228 reflections with *I* > 2σ(*I*)
graphite	*R*_int_ = 0.022
ω scans	θ_max_ = 31.3°, θ_min_ = 2.2°
Absorption correction: multi-scan (*SADABS*; Sheldrick, 1996)	*h* = −11→11
*T*_min_ = 0.768, *T*_max_ = 0.956	*k* = −13→13
15490 measured reflections	*l* = −13→13

### Refinement

**Table:**

Refinement on *F*^2^	Primary atom site location: structure-invariant direct methods
Least-squares matrix: full	Secondary atom site location: difference Fourier map
*R*[*F*^2^ > 2σ(*F*^2^)] = 0.040	Hydrogen site location: inferred from neighbouring sites
*wR*(*F*^2^) = 0.119	H atoms treated by a mixture of independent and constrained refinement
*S* = 1.01	*w* = 1/[σ^2^(*F*_o_^2^) + (0.0582*P*)^2^ + 0.1353*P*] where *P* = (*F*_o_^2^ + 2*F*_c_^2^)/3
3960 reflections	(Δ/σ)_max_ = 0.001
167 parameters	Δρ_max_ = 0.31 e Å^−^^3^
0 restraints	Δρ_min_ = −0.31 e Å^−^^3^

### Special details

**Table:**

Geometry. All e.s.d.'s (except the e.s.d. in the dihedral angle between two l.s. planes) are estimated using the full covariance matrix. The cell e.s.d.'s are taken into account individually in the estimation of e.s.d.'s in distances, angles and torsion angles; correlations between e.s.d.'s in cell parameters are only used when they are defined by crystal symmetry. An approximate (isotropic) treatment of cell e.s.d.'s is used for estimating e.s.d.'s involving l.s. planes.
Refinement. Refinement of *F*^2^ against ALL reflections. The weighted *R*-factor *wR* and goodness of fit *S* are based on *F*^2^, conventional *R*-factors *R* are based on *F*, with *F* set to zero for negative *F*^2^. The threshold expression of *F*^2^ > σ(*F*^2^) is used only for calculating *R*-factors(gt) *etc*. and is not relevant to the choice of reflections for refinement. *R*-factors based on *F*^2^ are statistically about twice as large as those based on *F*, and *R*- factors based on ALL data will be even larger.

### Fractional atomic coordinates and isotropic or equivalent isotropic displacement parameters (Å^2^)

**Table:**

	*x*	*y*	*z*	*U*_iso_*/*U*_eq_	
C1	0.19429 (17)	0.98956 (14)	0.61966 (13)	0.0409 (2)	
C2	0.1595 (2)	1.10035 (15)	0.72614 (15)	0.0470 (3)	
H2	0.1207	1.0779	0.8210	0.056*	
C3	0.1827 (2)	1.24378 (17)	0.69056 (18)	0.0563 (3)	
H3	0.1595	1.3170	0.7626	0.068*	
C4	0.2392 (3)	1.2813 (2)	0.5513 (2)	0.0695 (5)	
H4	0.2547	1.3783	0.5292	0.083*	
C5	0.2722 (3)	1.1734 (2)	0.44619 (19)	0.0675 (4)	
H5	0.3085	1.1987	0.3517	0.081*	
C6	0.2529 (2)	1.02670 (18)	0.47720 (15)	0.0503 (3)	
C7	0.2926 (3)	0.9189 (3)	0.35857 (18)	0.0692 (5)	
H7	0.3264	0.9554	0.2677	0.083*	
C8	0.36455 (18)	0.70690 (14)	0.85278 (13)	0.0413 (3)	
C9	0.5123 (2)	0.57064 (16)	0.79634 (16)	0.0519 (3)	
H9	0.4963	0.5055	0.7325	0.062*	
C10	0.6827 (2)	0.5337 (2)	0.83658 (19)	0.0628 (4)	
H10	0.7829	0.4425	0.7999	0.075*	
C11	0.7062 (2)	0.6304 (2)	0.93052 (19)	0.0621 (4)	
H11	0.8225	0.6047	0.9563	0.074*	
C12	0.5587 (2)	0.7649 (2)	0.98675 (18)	0.0599 (4)	
H12	0.5758	0.8296	1.0503	0.072*	
C13	0.3853 (2)	0.80416 (16)	0.94909 (15)	0.0487 (3)	
H13	0.2847	0.8941	0.9877	0.058*	
N1	0.16942 (19)	0.84378 (14)	0.64708 (14)	0.0509 (3)	
O1	0.00479 (14)	0.85915 (13)	0.90061 (12)	0.0576 (3)	
O2	0.13222 (17)	0.60899 (13)	0.76162 (14)	0.0649 (3)	
O3	0.2861 (2)	0.78653 (18)	0.36620 (14)	0.0795 (4)	
S1	0.14948 (5)	0.75289 (4)	0.79852 (4)	0.04575 (11)	
H1	0.199 (3)	0.790 (2)	0.5767 (19)	0.064 (5)*	

### Atomic displacement parameters (Å^2^)

**Table:**

	*U*^11^	*U*^22^	*U*^33^	*U*^12^	*U*^13^	*U*^23^
C1	0.0388 (6)	0.0369 (5)	0.0461 (6)	−0.0096 (4)	−0.0123 (5)	−0.0046 (5)
C2	0.0562 (7)	0.0365 (6)	0.0482 (7)	−0.0149 (5)	−0.0113 (6)	−0.0055 (5)
C3	0.0638 (9)	0.0377 (6)	0.0699 (9)	−0.0173 (6)	−0.0180 (7)	−0.0056 (6)
C4	0.0839 (12)	0.0498 (8)	0.0803 (11)	−0.0310 (8)	−0.0182 (10)	0.0112 (8)
C5	0.0769 (11)	0.0679 (10)	0.0581 (9)	−0.0294 (9)	−0.0116 (8)	0.0132 (8)
C6	0.0475 (7)	0.0533 (8)	0.0471 (7)	−0.0125 (6)	−0.0117 (6)	−0.0036 (6)
C7	0.0711 (10)	0.0802 (12)	0.0485 (8)	−0.0171 (9)	−0.0081 (7)	−0.0160 (8)
C8	0.0431 (6)	0.0316 (5)	0.0466 (6)	−0.0155 (4)	0.0004 (5)	0.0003 (4)
C9	0.0505 (7)	0.0380 (6)	0.0589 (8)	−0.0127 (5)	0.0027 (6)	−0.0044 (5)
C10	0.0474 (7)	0.0506 (8)	0.0725 (10)	−0.0056 (6)	0.0036 (7)	0.0057 (7)
C11	0.0481 (7)	0.0680 (10)	0.0680 (9)	−0.0209 (7)	−0.0118 (7)	0.0184 (8)
C12	0.0638 (9)	0.0618 (9)	0.0604 (9)	−0.0276 (7)	−0.0175 (7)	0.0038 (7)
C13	0.0518 (7)	0.0414 (6)	0.0511 (7)	−0.0160 (5)	−0.0062 (6)	−0.0039 (5)
N1	0.0662 (7)	0.0391 (5)	0.0508 (6)	−0.0199 (5)	−0.0134 (5)	−0.0108 (5)
O1	0.0440 (5)	0.0534 (6)	0.0689 (7)	−0.0164 (4)	0.0051 (5)	−0.0129 (5)
O2	0.0657 (7)	0.0436 (5)	0.0938 (8)	−0.0315 (5)	−0.0089 (6)	−0.0106 (5)
O3	0.0860 (9)	0.0802 (9)	0.0672 (8)	−0.0192 (7)	−0.0129 (7)	−0.0333 (7)
S1	0.04451 (18)	0.03489 (16)	0.0591 (2)	−0.01869 (12)	−0.00239 (14)	−0.00769 (12)

### Geometric parameters (Å, °)

**Table:**

C1—C2	1.3900 (17)	C8—C9	1.3871 (18)
C1—N1	1.3957 (17)	C8—S1	1.7515 (14)
C1—C6	1.4052 (19)	C9—C10	1.375 (2)
C2—C3	1.3797 (19)	C9—H9	0.93
C2—H2	0.93	C10—C11	1.374 (3)
C3—C4	1.375 (2)	C10—H10	0.93
C3—H3	0.93	C11—C12	1.376 (2)
C4—C5	1.365 (3)	C11—H11	0.93
C4—H4	0.93	C12—C13	1.383 (2)
C5—C6	1.390 (2)	C12—H12	0.93
C5—H5	0.93	C13—H13	0.93
C6—C7	1.453 (2)	N1—S1	1.6265 (13)
C7—O3	1.208 (2)	N1—H1	0.798 (18)
C7—H7	0.93	O1—S1	1.4212 (10)
C8—C13	1.3838 (18)	O2—S1	1.4241 (10)
			
C2—C1—N1	123.02 (12)	C10—C9—C8	118.75 (15)
C2—C1—C6	118.86 (12)	C10—C9—H9	120.6
N1—C1—C6	118.10 (12)	C8—C9—H9	120.6
C3—C2—C1	119.78 (14)	C11—C10—C9	120.54 (15)
C3—C2—H2	120.1	C11—C10—H10	119.7
C1—C2—H2	120.1	C9—C10—H10	119.7
C4—C3—C2	121.69 (15)	C10—C11—C12	120.40 (15)
C4—C3—H3	119.2	C10—C11—H11	119.8
C2—C3—H3	119.2	C12—C11—H11	119.8
C5—C4—C3	118.78 (15)	C11—C12—C13	120.29 (16)
C5—C4—H4	120.6	C11—C12—H12	119.9
C3—C4—H4	120.6	C13—C12—H12	119.9
C4—C5—C6	121.51 (16)	C12—C13—C8	118.66 (14)
C4—C5—H5	119.2	C12—C13—H13	120.7
C6—C5—H5	119.2	C8—C13—H13	120.7
C5—C6—C1	119.37 (14)	C1—N1—S1	128.49 (9)
C5—C6—C7	117.69 (16)	C1—N1—H1	112.3 (14)
C1—C6—C7	122.94 (15)	S1—N1—H1	116.5 (13)
O3—C7—C6	126.49 (17)	O1—S1—O2	119.76 (7)
O3—C7—H7	116.8	O1—S1—N1	109.04 (7)
C6—C7—H7	116.8	O2—S1—N1	103.70 (7)
C13—C8—C9	121.35 (13)	O1—S1—C8	108.57 (7)
C13—C8—S1	120.66 (10)	O2—S1—C8	108.83 (7)
C9—C8—S1	117.98 (11)	N1—S1—C8	106.08 (6)
			
N1—C1—C2—C3	−178.37 (13)	C9—C10—C11—C12	0.6 (2)
C6—C1—C2—C3	0.1 (2)	C10—C11—C12—C13	−0.1 (2)
C1—C2—C3—C4	0.2 (2)	C11—C12—C13—C8	−0.9 (2)
C2—C3—C4—C5	0.3 (3)	C9—C8—C13—C12	1.3 (2)
C3—C4—C5—C6	−1.1 (3)	S1—C8—C13—C12	−178.76 (11)
C4—C5—C6—C1	1.4 (3)	C2—C1—N1—S1	−16.6 (2)
C4—C5—C6—C7	−179.47 (18)	C6—C1—N1—S1	164.92 (11)
C2—C1—C6—C5	−0.8 (2)	C1—N1—S1—O1	52.78 (14)
N1—C1—C6—C5	177.67 (14)	C1—N1—S1—O2	−178.56 (12)
C2—C1—C6—C7	−179.97 (14)	C1—N1—S1—C8	−63.97 (14)
N1—C1—C6—C7	−1.4 (2)	C13—C8—S1—O1	−19.79 (13)
C5—C6—C7—O3	178.68 (18)	C9—C8—S1—O1	160.20 (10)
C1—C6—C7—O3	−2.2 (3)	C13—C8—S1—O2	−151.70 (11)
C13—C8—C9—C10	−0.7 (2)	C9—C8—S1—O2	28.29 (13)
S1—C8—C9—C10	179.30 (11)	C13—C8—S1—N1	97.27 (11)
C8—C9—C10—C11	−0.2 (2)	C9—C8—S1—N1	−82.73 (11)

### Hydrogen-bond geometry (Å, °)

**Table:**

*D*—H···*A*	*D*—H	H···*A*	*D*···*A*	*D*—H···*A*
N1—H1···O3	0.80 (2)	1.99 (2)	2.6751 (19)	144 (2)
C2—H2···O1	0.93	2.46	3.0879 (18)	125
C3—H3···O2^i^	0.93	2.56	3.2691 (19)	133
C5—H5···Cg1^ii^	0.93	2.80	3.700 (2)	162

Symmetry codes: (i) *x*, *y*+1, *z*; (ii) −*x*+1, −*y*+2, −*z*+1.
